# β-globin haplotypes in normal and hemoglobinopathic individuals from Reconcavo Baiano, State of Bahia, Brazil

**DOI:** 10.1590/S1415-47572010005000042

**Published:** 2010-09-01

**Authors:** Wellington dos Santos Silva, Maria de Nazaré Klautau-Guimarães, Cesar Koppe Grisolia

**Affiliations:** 1 Faculdade Adventista da Bahia, Cachoeira, BABrazil; 2 Departamento de Genética e Morfologia, Instituto de Ciências Biológicas, Universidade de Brasília, Brasília, DFBrazil

**Keywords:** Afro-descendant, [##946, ]-globin, haplotypes, hemoglobinopathies

## Abstract

Five restriction site polymorphisms in the β-globin gene cluster (*HincII-5*‘ ε, *HindIII-*^*G*^ γ, *HindIII-*^*A*^ γ, *HincII-* ψβ*1* and *HincII-3*‘ ψβ*1*) were analyzed in three populations (n = 114) from Reconcavo Baiano, State of Bahia, Brazil. The groups included two urban populations from the towns of Cachoeira and Maragojipe and one rural Afro-descendant population, known as the “quilombo community”, from Cachoeira municipality. The number of haplotypes found in the populations ranged from 10 to 13, which indicated higher diversity than in the parental populations. The haplotypes 2 (+ - - - -), 3 (- - - - +), 4 (- + - - +) and 6 (- + + - +) on the β^A^ chromosomes were the most common, and two haplotypes, 9 (- + + + +) and 14 (+ + - - +), were found exclusively in the Maragojipe population. The other haplotypes (1, 5, 9, 11, 12, 13, 14 and 16) had lower frequencies. Restriction site analysis and the derived haplotypes indicated homogeneity among the populations. Thirty-two individuals with hemoglobinopathies (17 sickle cell disease, 12 HbSC disease and 3 HbCC disease) were also analyzed. The haplotype frequencies of these patients differed significantly from those of the general population. In the sickle cell disease subgroup, the predominant haplotypes were BEN (Benin) and CAR (Central African Republic), with frequencies of 52.9% and 32.4%, respectively. The high frequency of the BEN haplotype agreed with the historical origin of the afro-descendant population in the state of Bahia. However, this frequency differed from that of Salvador, the state capital, where the CAR and BEN haplotypes have similar frequencies, probably as a consequence of domestic slave trade and subsequent internal migrations to other regions of Brazil.

## Introduction

For more than 20 years haplotypes from the 5' region of the β-globin gene cluster have been used to study the origin and distribution of human populations (Wainscoat *et al.*, 1986), as well as to trace the gene flux of variant hemoglobins HbS and HbC from Africa to other continents (Antonarakis *et al.*, 1984; Pagnier *et al.*, 1984).

The β-globin gene cluster system has proven to be very useful in studies of the evolutionary relationships of African, Eurasian and Pacific Islander populations. The results of these studies have supported an African origin for modern *Homo sapiens* and have helped to determine the major patterns of β-globin gene distribution. On a global scale, haplotypes 2 (+ - - - -), 5 (- + - + +) and 6 (- + + - +) are the most prevalent in Eurasians whereas in Africans haplotype 3 (- - - - +) reaches a frequency ≥ 50% (Wainscoat *et al.*, 1986; Long *et al.*, 1990; Chen *et al.*, 1990). Guerreiro *et al.* (1992) and Bevilaqua *et al.* (1995) reported the distribution of these haplotypes among Brazilian Indians.

In Brazil, the first study of β-globin haplotypes among patients with sickle cell anemia found the three most common haplotypes linked to the β^S^ mutation from the African continent. Of 67 chromosomes, 49 (66.2%) had the Central African Republic (CAR) haplotype, 17 (23.0%) had the Benin (BEN) haplotype and one (1.3%) had the Senegal (SEN) haplotype (Zago *et al.*, 1992).

A subsequent study examined haplotypes of the β^S^ cluster in 74 patients with sickle cell anemia from three representative towns in the regions with the highest number of afro-descendants in Brazil: Ribeirão Preto (State of São Paulo) in the southeast of Brazil, Salvador (State of Bahia) in the northeast and Belém (State of Pará) in the north (Figueiredo *et al.*, 1994). The three most common African haplotypes were found in 138 chromosomes: the CAR haplotype predominated in the three regions (73.1% in São Paulo, 54.8% in Bahia and 65.9% in Pará), followed by the BEN haplotype (25.4% in São Paulo, 45.2% in Bahia and 27.6% in Pará) and a small number of cases of the SEN haplotype in São Paulo (1.5%) and Pará (6.9%). Figueiredo *et al.* (1996) later reported frequencies of 61.8% and 34.7% for the CAR and BEN haplotypes, respectively, in the State of São Paulo.

**Figure 1:**
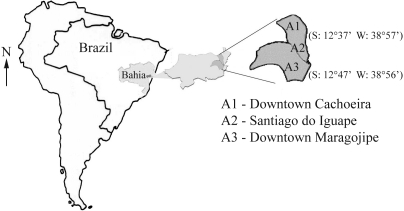
Map showing the locations of the towns in the Reconcavo Baiano studied in this work. A1 - Downtown Cachoeira, A2 - Santiago do Iguape, A3 - Downtown Maragojipe.

Reconcavo Baiano, the focus of our study, is the region surrounding a large bay on the Atlantic Coast of Brazil, bordered to the north by the state capital Salvador (Figure 1). This region, which includes many historic and economically important cities, has had a long, close association with the state capital and with the history of the African slave trade. As a result, the Reconcavo Baiano has many Afro-derived Brazilian populations or “quilombos” that were originally founded by runaway slaves (Alencastro, 2000).

Although DNA polymorphisms in the 5' region of the β-globin cluster have been reported in populations from the state of Bahia (Figueiredo *et al.*, 1994; Gonçalves *et al.*, 2003; Adorno *et al.*, 2004; Lyra *et al.*, 2005), most of these studies have focused on the urban population of Salvador. There is no study of genetic variability in Afro-descendant populations from the Reconcavo Baiano region. Thus, the main goal of this study was to characterize the haplotypes resulting from DNA polymorphisms in the 5' region of the β-globin cluster and their distribution in populations from the Reconcavo Baiano. We also used these data to analyze intra- and interpopulational variability and genetic mixture in order to estimate the contribution of parental populations to the gene pool of the Reconcavo Baiano region.

## Material and Methods

The sample for this study consisted of 114 unrelated male and female individuals from the towns of Cachoeira (S: 12°37'04" W: 38°57'21") and Maragojipe (S: 12°47' W: 38°56'). Of the 48 individuals living in central Maragojipe, 44 had HbAA, 1 had HbAC and 3 had HbAS electrophoretic profiles. The subjects from Cachoeira consisted of two groups: one of 34 individuals living in the town center (30 HbAA, 1 HbAC and 3 HbAS) and the other of 32 individuals from the Afro-derived population of Santiago do Iguape (26 HbAA 1 HbAC and 5 HbAS), a village located 44 km from central Cachoeira. These individuals were selected in community health programs run by the Faculdade Adventista da Bahia in partnership with the Fourth Regional Health Division of the State of Bahia and local health boards. The programs offer various health services such as vaccination, prenatal supervision, sex education, dental treatment and blood pressure and glycemia measurements.

In addition to these subjects, a further 22 individuals (male and female) bearing the sickle-cell trait (HbAS) and 32 unrelated patients with hemoglobinopathies (17 sickle-cell disease, 12 HbSC disease and 3 HbCC disease) diagnosed in the Genetics Laboratory of the Department of Physiotherapy at the Faculdade Adventista were selected from other towns in the region.

Blood samples (5 mL) for electrophoretic and molecular analyses were collected in tubes containing EDTA (0.03%) as anticoagulant. DNA from leukocytes in 100 μL of venous blood was isolated using GFX Genomic Blood DNA purification kits (Amersham Lifesciences, Piscataway, NJ, USA). The presence of the variant hemoglobins HbS and HbC detected by the electrophoresis of hemoglobin in alkaline medium was confirmed by the polymerase chain reaction (PCR) followed by digestion with an appropriate restriction enzyme. The reaction protocol was a modified version of the Amplification Refractory Mutation System (ARMS) described by Attila *et al.* (2004).

DNA polymorphism was assessed by using a slightly modified version of the technique proposed by Sutton *et al.* (1989). Initially, DNA segments containing one of each of the polymorphic sites that we analyzed, namely, 1) *Hinc*II, in the 5' region of the ε gene, 2) *Hind*III in IVS-2 of the ^G^γ gene, 3) *Hind*III in IVS-2 of the ^A^γ gene, 4) *Hinc*II in the ψβ gene and 5) *Hinc*II in the 3' region of the ψβ gene, were amplified by PCR. The amplifications were done in a final reaction volume of 25 μL containing 25 pM of each primer, 10 mM Tris-HCl (pH 8.3), 50 mM KCl, 1.5 mM MgCl_2_, 200 μM of each deoxynucleotide triphosphate (dATP, dCTP, dGTP, dTTP) (Amersham Lifesciences), 1.0 U of *Taq* polymerase (Perkin Elmer Cetus Corporation, Norwalk, USA) and 100 ng of genomic DNA. The reactions consisted of an initial step of DNA denaturation at 95 °C for 2 min, followed by 35 cycles of 1 min at 94 °C for denaturation, 1 min at 54-57 °C for primer pairing, 2 min at 72 °C for polymerization and a final step of 7 min at 72 °C. The amplified products were digested with appropriate restriction enzymes and visualized in 1.5% agarose gels stained with ethidium bromide (0.5 μg/mL); φX174 DNA digested with *Hae*III was used as the size ladder.

Haplotypes and their respective frequencies were identified with Phase 2.1.1, a computer program that uses Bayesian algorithms (Stephens and Scheet, 2005). Inter-population genetic diversity was analyzed by the G_ST_ calculation as provided by Fstat software based on equation 8.27 described by Nei (1987). This estimate was obtained in order to allow comparison with data in the literature since several recent reports have used G_ST_ in their analyses of inter-populational diversity. Ethnic admixture was estimated by the gene identification method (Chakraborty, 1985) using the program ADMIX95 for a three-hybrid population. Representative allelic frequencies for African, European and native populations agreed with the mean frequencies reported in the current literature. This project was approved by the Ethics Committee of the University of Brasilia (protocol no. 021.0.000.012-04).

## Results

Table 1 compares the distribution frequencies of the restriction sites in the β-globin gene complex of the three samples with those of the parental populations. The software Phase 2.1.1 identified 22 haplotypes in the β-globin cluster that had a frequency ≥ 1%, based on a non-recombination method. Fourteen of these haplotypes were linked to β^A^ chromosomes, six to β^S^ chromosomes and two to β^C^ chromosomes.

The haplotypes linked to β^A^ chromosomes most commonly encountered in the samples from Maragojipe, Cachoeira and Iguape were 2, 3, 4, and 6 (Table 2). In addition, two haplotypes, 9 and 14, were specific to the Maragojipe population. Seven haplotypes identified in the three p opulations (haplotypes 1, 2, 3, 4, 5, 6 and 16) were considered first order haplotypes that originated by point mutations or gene-conversion events; haplotype 2 is likely to have been the ancestral form. Haplotypes 9, 11, 12, 13 and 14 were considered second order haplotypes and may have originated from recombination among first order haplotypes, as suggested in a scheme proposed by Long *et al.* (1990) and Chen *et al.* (1990).

The haplotype diversity indices obtained for the Maragojipe, Cachoeira and Iguape populations were 0.899, 0.870 and 0.835, respectively. The variability for the haplotype diversity indices (Hs and Ht) and the gene differentiation coefficients (Gst and Gst') for the populations from Reconcavo Baiano and other ethnic groups are shown in Table 3.

The extent of ethnic racial admixture was assessed by considering the three populations as a single sample. A three-hybrid population model was used to estimate the percentages for each parental population and yielded the following results: 80.4% African, 10.8% European and 8.8% native Amerindian, with a significant coefficient for multiple correlation (R^2^ = 0.99).

Table 4 shows the haplotype distribution among hemoglobinopathic patients. BEN (Benin - Central West Africa) and CAR (Central African Republic) were the dominant haplotypes in β^S^ chromosomes, with frequencies of 52.9% and 32.4%, respectively, in the sickle cell disease group; types I and II were the dominant haplotypes in β^C^ chromosomes, with frequencies of 55.5% and 44.5%, respectively. Table 5 shows the frequencies for the β^S^ haplotypes in some Brazilian populations compared to those found in populations from the Reconcavo Baiano region. The frequency of the BEN haplotype was greater in the states of Bahia and Ceará, while in the other states the CAR haplotype was more frequent. The presence of the type-I haplotype (- + - - +) in β^C^ chromosomes agreed with historical data ascribing the origin of this haplotype to Central and West Africa, a region from which large numbers of slaves came to Brazil, especially to northeastern Brazil. However, the frequency of the type II haplotype in the Reconcavo Baiano region was higher than in African populations (Boehm *et al.*, 1985; Talacki *et al.*, 1990).

## Discussion

Analysis of the distribution frequencies of the restriction sites showed that the frequencies for the *Hinc*II 5'ε site (0.217 to 0.325) were intermediate between those of the African population (0.108) and those of the European (0.618) and Amerindian (0.81) populations (Table 1). For the *Hind*III IVS2 ^G^γ site, the frequencies of the samples from Maragojipe (0.436) and Iguape (0.453) were closer to those of the African population (0.463), whereas the Cachoeira sample had a lower frequency (0.323). Maragojipe (0.260) and Iguape (0.196) showed the highest frequencies for the *Hind*III IVS2 ^A^γ restriction site, and the frequency for Cachoeira (0.088) was similar to that of the African population (0.079). The frequency of the *Hinc*II ψβ1 site in the Iguape sample (0.018) was closer to the Amerindian population (0.04). The latter data indicated only a distant relationship (based on frequency) with the Maragojipe (0.156) and Cachoeira (0.100) populations, which were closer to the African population (0.158). The *Hinc*II 3'ψβ1 site had the lowest variability among restriction sites in the three samples (0.633 and 0.672), with values between those of the European (0.359) and African (0.931) populations.

Fourteen haplotypes identified in the three populations accounted for 43.7% of the 32 possible haplotypes. This linkage-disequilibrium resulted from the proximity among restriction sites in a region of ~32 kb where the overall recombination rate was estimated at 0.0017% (Chakravarti *et al.*, 1984; Wood *et al.*, 2005). Recombination of the most common haplotypes allowed for the existence of two atypical haplotypes.

Table 2 compares the haplotype frequencies of the three populations studied with the parental populations. The frequencies of haplotype 2 in the three samples were much higher than in the African population (0.063), and also greater than for the European and Amerindian populations (0.609 and 0.843, respectively). In contrast, the frequency of haplotype 3 in the three samples was, at the most, half of the frequency found in the African population (0.532). The frequencies for haplotype 4 in the Maragojipe (0.1364) and Cachoeira (0.1667) populations were similar to that in the African population (0.152), whereas the Iguape population had the highest frequency for this haplotype (0.2308).

Haplotypes 18 and 19 were first described in Japanese subjects by Shimizu *et al.* (1992). Haplotype 18 was also subsequently identified in a mixed-blood Mexican population (Villalobos-Arambula *et al.*, 1997) and in Colombian Amerindians (Shimizu *et al.*, 2001). This haplotype had frequencies of 0.0682 and 0.0167 in the Maragojipe and Cachoeira populations, respectively, and was not detected in the Iguape population. Haplotype 19 was found in the populations of Maragojipe (0.0341) and Iguape (0.0192). However, there is no evidence of these two haplotypes in African, European and Brazilian Amerindian populations (Mousinho-Ribeiro *et al.*, 2003; Callegari-Jacques *et al.*, 2007).

The low coefficient of gene differentiation (Gst) for the three populations from Reconcavo Baiano (0.004; Table 3) indicated that the quilombo population from Santiago do Iguape shared no genetic structure with the urban populations of Cachoeira and Maragojipe. interethnic admixture may be the most important factor in the higher variability observed among the populations from Reconcavo Baiano. The ethnic admixture observed here agreed with the morphological data reported elsewhere (Azevedo, 1980), and with the outcomes of autosomal microsatellites recorded in the Bananal community, a village that was started by runaway slaves near the city of Jequié in Bahia (Barbosa *et al.*, 2006). Although the population of this community has a marked African influence, interethnic contacts throughout the community's history have had a profound impact on its genetic makeup.

Studies of the β^S^ haplotypes among HbSS individuals in northern, northeastern and southern Brazil have indicated that the average frequency for the predominant CAR haplotype in the three regions (65.9%) agrees with historical data showing that about 70% of the African slaves transported to Brazil were from Angola, the Congo and Mozambique (Zago *et al.*, 1992; Figueiredo *et al.*, 1994). Bezerra *et al.* (2007) found a higher frequency for the CAR haplotype (79.2%) in Pernambuco state, while the opposite was observed for the BEN haplotype (15.1%). Comparison of these results with those for other states in northeastern Brazil indicates that the African descendants in this region have a heterogeneous origin.

Two studies have examined HbSS individuals from Salvador: in one, 17 out of 80 patients had the CAR/CAR genotype (21.3%) (Gonçalves *et al.*, 2003), while in the other, eight out of 40 patients (20.0%) had this genotype (Lyra *et al.*, 2005). In contrast, the frequency of this genotype did not exceed 12.0% among individuals in Reconcavo Baiano. In a more recent study involving a larger sample of patients with sickle cell anemia in the city of Salvador, 14.4% had the CAR/CAR genotype (Adorno *et al.*, 2008). This phenomenon can be explained by internal migrations of Afro-descendants from other Brazilian regions to the State capital. Cardoso and Guerreiro (2006) observed the same phenomenon when they studied haplotype distribution in HbSS individuals from Belém city, in northern Brazil; these authors also found a higher frequency for the CAR haplotype than reported in historical records.

The Cameroon (CAM) haplotype that we identified comes from the West African coast, especially Nigeria (Kulozik *et al.*, 1986; Oner *et al.*, 1992). Other studies have also identified this haplotype in northeastern Brazil (Adorno *et al.*, 2004, 2008; Bezerra *et al.*, 2007). Cardoso and Guerreiro (2006) described this haplotype when they analyzed the population in Belém and attributed it to slave migrations from northeastern to northern Brazil.

The historian Pierre Verger stated that the Nagô-Ioruba influence in the State of Bahia originated with slaves brought to that state from the Gulf of Benin region. In contrast, other Brazilian states received most of their slaves from the Congo and Angola, where the CAR haplotype dominates (Verger, 1968). In the late 16^th^ and early 17^th^ centuries, there was intense slave trade from harbors in Ghana and Nigeria in the Gulf of Benin to Salvador and Recife, with several Afro-Brazilian religions being based on religious practices from this region in West Africa, where the Ioruba group predominates (Klein, 2002). In the 19^th^ century, when slavery became illegal in Brazil, most of the slaves who were manumitted in Salvador came from West Africa (Nishida, 1993).

The frequencies of the type I and type II β^C^ haplotypes found in Reconcavo Baiano (55.50% and 45.50%, respectively) differ from those reported by Bezerra *et al.* (2007) for Pernambuco state where the frequency of the type I haplotype was 80.0%, and those of the type II and type III haplotypes were 13.2% and 6.6%, respectively. Thus, the results from Pernambuco more closely resemble those found in African populations.

The frequency of 45.5% for the type II haplotype (- - - - +) in β^C^ chromosomes was higher than in Africa, where it ranges from 8% to 23% (Boehm *et al.*, 1985; Talacki *et al.*, 1990). One possible explanation for the increase in this haplotype could be a bottleneck or founding effect. In this scenario, individuals who formed the first population that settled the Reconcavo Baiano region probably differed in their frequencies of both β^C^ haplotypes when compared to the ancestral populations, and this could have led to an increase in the frequency of the type II β^C^ haplotype.

## Figures and Tables

**Table 1:** Frequencies of restriction sites in the β-globin gene complex and in the parental populations (African, Amerindian and European) of the samples studied.

Population	*Hinc*II	*Hind*III	*Hind*III	*Hinc*II	*Hinc*II
	*5*'ε	*IVS2*^*G*^γ	*IVS2*^*A*^γ	ψβ*1*	*3*‘ψβ*1*
Maragojipe	0.325	0.436	0.26	0.156	0.66
Cachoeira	0.317	0.323	0.088	0.10	0.633
Iguape	0.217	0.453	0.196	0.018	0.672
African (center-South)^*^	0.108	0.463	0.079	0.158	0.931
European^*^	0.618	0.379	0.15	0.222	0.359
Amerindian^#^	0.81	0.18	0.13	0.04	0.15

*Data from Wainscoat *et al.* (1986) and Long *et al.* (1990). ^#^Data from Guerreiro *et al.* (1994) and Bevilaqua *et al.* (1995).

**Table 2:** Frequencies of the haplotypes connected to the β^A^ chromosome in the samples studied and the parental populations.

Haplotypes* (number of chromosomes)	Maragojipe (88)	Cachoeira (60)	Iguape (52)	African^#^ (79)	European^#^ (258)	Amerindian^§^ (477)
1. -----	0.0795	0.1000	0.0577	0.0000	0.0000	0.0060
2. +----	0.1591	0.2000	0.1346	0.063	0.609	0.843
3. ----+	0.1477	0.2167	0.2692	0.532	0.019	0.0000
4. -+--+	0.1364	0.1667	0.2308	0.152	0.0000	0.0060
5. -+-++	0.0795	0.0500	0.0000	0.139	0.377	0.0060
6. -++-+	0.1364	0.0833	0.1346	0.025	0.113	0.122
9. -++++	0.0341	0.0000	0.0000	0.0000	0.0000	0.0120
11. ---++	0.0455	0.0333	0.0192	0.0000	0.0000	0.0000
12. ++---	0.0455	0.0000	0.0577	0.0000	0.0000	0.0000
13. +---+	0.0227	0.1000	0.0192	0.038	0.0000	0.0000
14. ++--+	0.0114	0.0000	0.0000	0.013	0.0000	0.0000
16. -+---	0.0000	0.0333	0.0577	0.013	0.0000	0.0000
18. +-+--	0.0682	0.0167	0.0000	0.0000	0.0000	0.0000
19.--+-+	0.0341	0.0000	0.0192	0.0000	0.0000	0.0000

*Haplotypes 1 to 16 numbered according to Long *et al.* (1990) and haplotypes 18 and 19 according to Shimizu *et al.* (1992, 2001) and Villalobos-Arambula *et al.* (1997). ^#^Data from Long *et al.* (1990) and Chen *et al.* (1990). ^§^Data from Guerreiro *et al.* (1992).

**Table 3:** Haplotype diversity for the β-globin cluster in populations from the Reconcavo Baiano area and parental populations.

Population	Number of subpopulations	Hs	Ht	Dst	Gst	Gst'
Reconcavo Baiano	3	0.868	0.872	0.004	0.004	0.006
African^*^	6	0.650	0.710	0.060	0.085	0.100
European^*^	5	0.590	0.820	0.030	0.280	0.060
Amerindian^#^	18	0.290	0.305	0.015	0.049	0.052

Hs, average heterozygosity within populations; Ht, average heterozygosity for the entire population; Dst, interpopulational genetic variation; Gst, coefficient of gene differentiation; Gst', coefficient of gene differentiation, considering the number of populations examined. *Data from Long *et al.* (1990). ^#^Data from Mousinho-Ribeiro *et al.* (2003).

**Table 4:** Haplotype distributions for β^S^ and β^C^ chromosomes in the three groups of hemoglobinopathic patients from the Reconcavo Baiano.

Haplotypes	Patients	Patients	Patients	Total
	HbSS	HbSC	HbCC	
β^S^ chromosome				
BEN	18	6	-	24
CAR	11	4	-	15
CAM	1	0	-	1
Atypical	4	2	-	6
Total	34	12	-	46
β^C^ chromosome				
Type I	-	9	1	10
Type II	-	3	5	8
Total	-	12	6	18

**Table 5:** Frequency distribution (%) for the β^S^ haplotypes in the Reconcavo Baiano area and other Brazilian populations.

Population	N	Haplotypes					
		CAR	BEN	SEN	CAM	ARAB	Atypical
Reconcavo Baiano	34	32.5	52.9	0.0	2.9	0.0	11.8
Salvador (BA)^1^	250	41.6	55.2	0.4	1.2	0.4	1.2
Ceará^2^	44	31.8	43.2	2.3	0.0	0.0	22.7
Pernambuco^3^	127	81.1	14.2	0.0	0.8	0.0	3.9
Belém (PA)^4^	260	66.0	21.8	10.9	1.3	0.0	0.0
São Paulo/Campinas (SP)^5^	142	64.8	35.2	0.0	0.0	0.0	0.0
Ribeirão Preto (SP)^6^	67	73.1	25.4	1.5	0.0	0.0	0.0
Porto Alegre (RS)^7^	49	79.6	18.4	2.0	0.0	0.0	0.0

N = number of chromosomes. 1. Adorno *et al.* (2008), 2. Galiza Neto *et al.* (2005), 3. Bezerra *et al.* (2007), 4. Cardoso and Guerreiro (2006), 5. Gonçalves *et al.* (1994), 6. Figueiredo *et al.* (1994), 7. Wagner *et al.* (1996).
